# Osseous Union of Teeth

**Published:** 1853-01

**Authors:** 


*Osseous Union of Teeth.*—Dr. S. P. Hullihen, of Wheeling, Va., recently sent to us for the Museum of the Baltimore College of Dental Surgery, a beautiful specimen of osseous union of a permanent central and lateral incisor of the upper jaw. This is the third or fourth example of osseous union of the crowns of teeth of second dentition which we have seen. With the temporary teeth, occurrences of this sort are more frequent. There are several examples of it in the museum of the above institution, but this is the only one there of osseous union of the crowns of permanent teeth.

Dr. V. M. Swayze, of Easton, Penn., says in a letter, which we recently received from him, that he extracted a few days before, an upper dens sapientiæ to which a supernumerary tooth of the size of a temporary cuspid was attached, throughout its whole length, by osseous union. The writer also adds that the corresponding tooth on the other side of the jaw, has a supernumerary united to it nearly as large as that of the other tooth, giving it the appearance of a double dens sapientiæ. We wish we could persuade his patient to have that extracted, and he be induced to present one of the specimens to the museum of the Baltimore College.

